# Mechanical thrombectomy – an alternative treatment option in a patient with acute ischemic stroke and multiple contraindications for systemic thrombolysis: a case report

**DOI:** 10.1186/1752-1947-7-256

**Published:** 2013-11-07

**Authors:** Katrin Christina Sczesni, Reinhard Wiebringhaus, Lothar Heuser, Sabine Skodda, Jens Eyding

**Affiliations:** 1 Department of Neurology, Knappschaftskrankenhaus, Ruhr University, In der Schornau 23-25, 44892 Bochum, Germany; 2 Department of Radiology, Knappschaftskrankenhaus, Ruhr University, In der Schornau 23-25, 44892 Bochum, Germany

**Keywords:** Acute ischemic stroke, Endovascular treatment, Mechanical thrombectomy, Thrombolysis

## Abstract

**Introduction:**

Acute ischemic stroke is a common cause of disability and death in developed countries. Standard therapy for patients who present within 4.5 hours from the onset of symptoms is intravenous thrombolysis if contraindications such as oral anticoagulation, cancer or recent surgery are ruled out. Apart from that, mechanical recanalization is a new treatment option for patients with occlusion of major cerebral arteries as a cause of ischemic stroke.

**Case presentation:**

In this case report we describe a 55-year-old Caucasian man with a right hemispheric ischemic syndrome who presented in time but who had multiple contraindications against systemic thrombolysis. He was then treated with mechanical recanalization and recovered. On discharge from the hospital he had only a slight left-sided facial paresis and discrete impairment of motion smoothness in his left hand.

**Conclusion:**

We conclude that multimodal imaging should be performed in all patients with an acute onset of neurological symptoms suspicious of ischemic stroke, even if they have contraindications against an intravenous thrombolytic treatment.

## Introduction

Acute ischemic stroke is a common cause of disability and death in developed countries. Standard therapy for patients who present within 4.5 hours from the onset of symptoms is intravenous thrombolysis if contraindications such as oral anticoagulation, cancer or recent surgery are ruled out [1-3]. Apart from that, mechanical recanalization is a promising new treatment option for patients with occlusion of major cerebral arteries as a cause of ischemic stroke. It is frequently used in addition to intravenous thrombolytic therapy (“bridging concept”) [4-6].

Here, we describe a patient with a right hemispheric ischemic syndrome who presented timely but who had multiple contraindications against systemic thrombolysis. He was then treated with mechanical recanalization.

## Case presentation

A 55-year-old Caucasian man presented to the emergency room because of an acute loss of vision “on the right side”. He could not differentiate between hemianopsia and amaurosis. During the ophthalmological examination he developed a head deviation to the right side, dysarthria, central left-sided facial paresis, a severe left-sided hemiparesis and neglect to the left (National Institutes of Health Stroke Scale, NIHSS, 13). He was under treatment for urothelial carcinoma of the bladder with vesicular Bacillus Calmette–Guérin installation every 6 weeks and tumor response was documented at the last control examinations. In addition to this, he had suffered two deep venous thromboses in the past because of thrombophilia with elevated levels for factor VIII. Therefore, he was on oral anticoagulation with phenprocoumon with an effective international normalized ratio of 2.25 on the day of admission.

**Figure 1:**
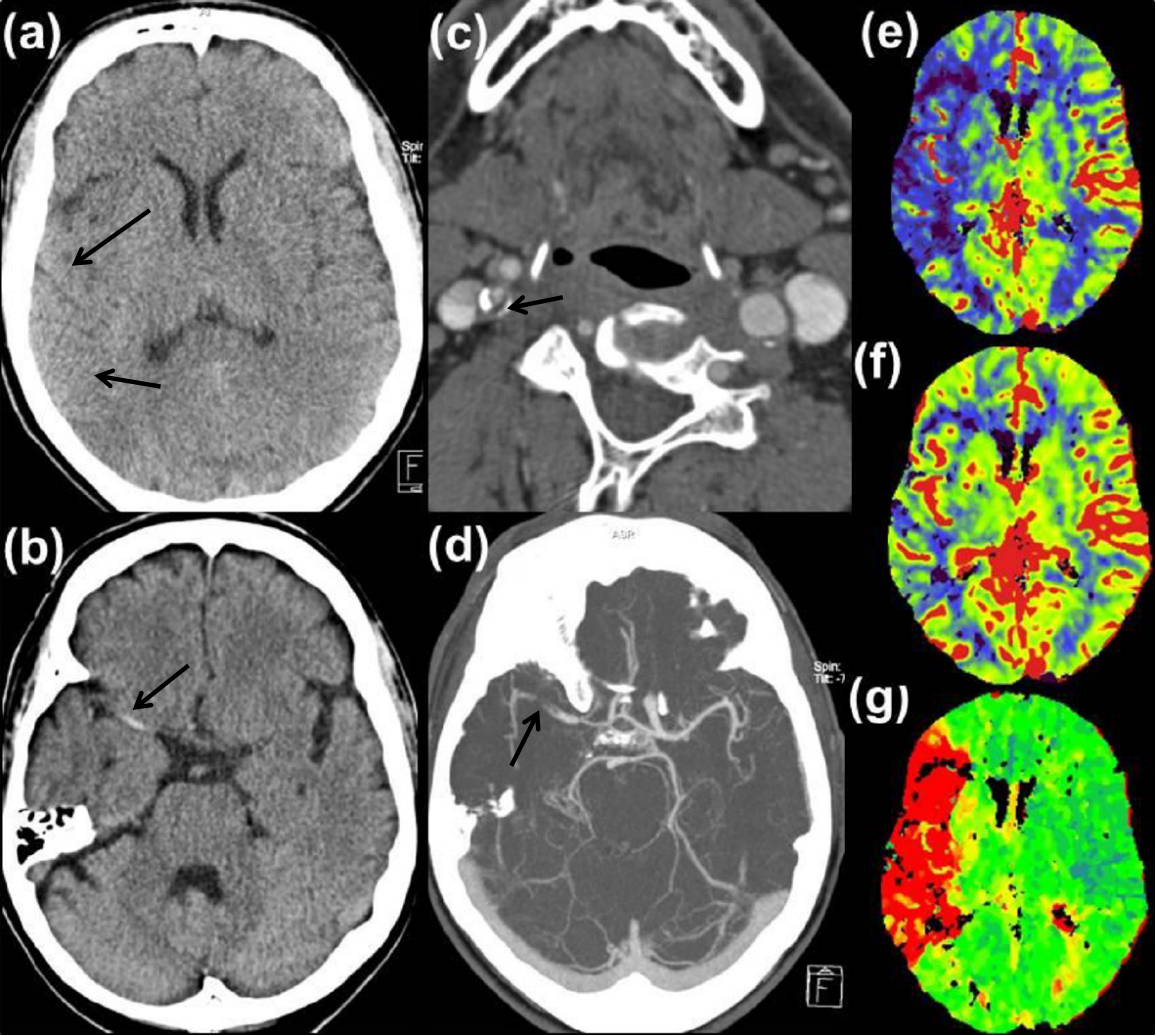
**Early signs of ischemia in native cranial computed tomography scan with effacement of sulci and loss of the insular ribbon in right middle cerebral artery territory (a) and hyperdense right middle cerebral artery sign (b).** High-grade calcified stenosis and thrombotic material in right internal carotid artery **(c)**. Computed tomography angiography displaying a fresh thrombus in right middle cerebral artery **(d)**. Computed tomography perfusion imaging with a clear perfusion deficit (red area of time-to-peak **(g)** and a significant mismatch of cerebral blood flow **(e)** to cerebral blood volume **(f)**.

Cranial multimodal computed tomography (CT) imaging following a standard protocol including CT angiography (CTA) and CT perfusion imaging (CTP) was performed immediately. Intracerebral hemorrhage was excluded. A “dense artery sign” with projection to his right middle cerebral artery (RMCA) and an incipient loss of differentiation between cortex and white matter in the insular cortex was detectable (Figure 1(a) and (b)). CTA revealed a high-grade stenosis of his right internal carotid artery (RICA; Figure 1(c)), along with thrombotic material in his RICA and in his RMCA (Figure 1(d)). CTP revealed a time-to-peak delay in the area of his RMCA with apparent mismatch between cerebral blood flow (CBF) and cerebral blood volume (CBV; Figure 1(e)-(g)).

**Figure 2:**
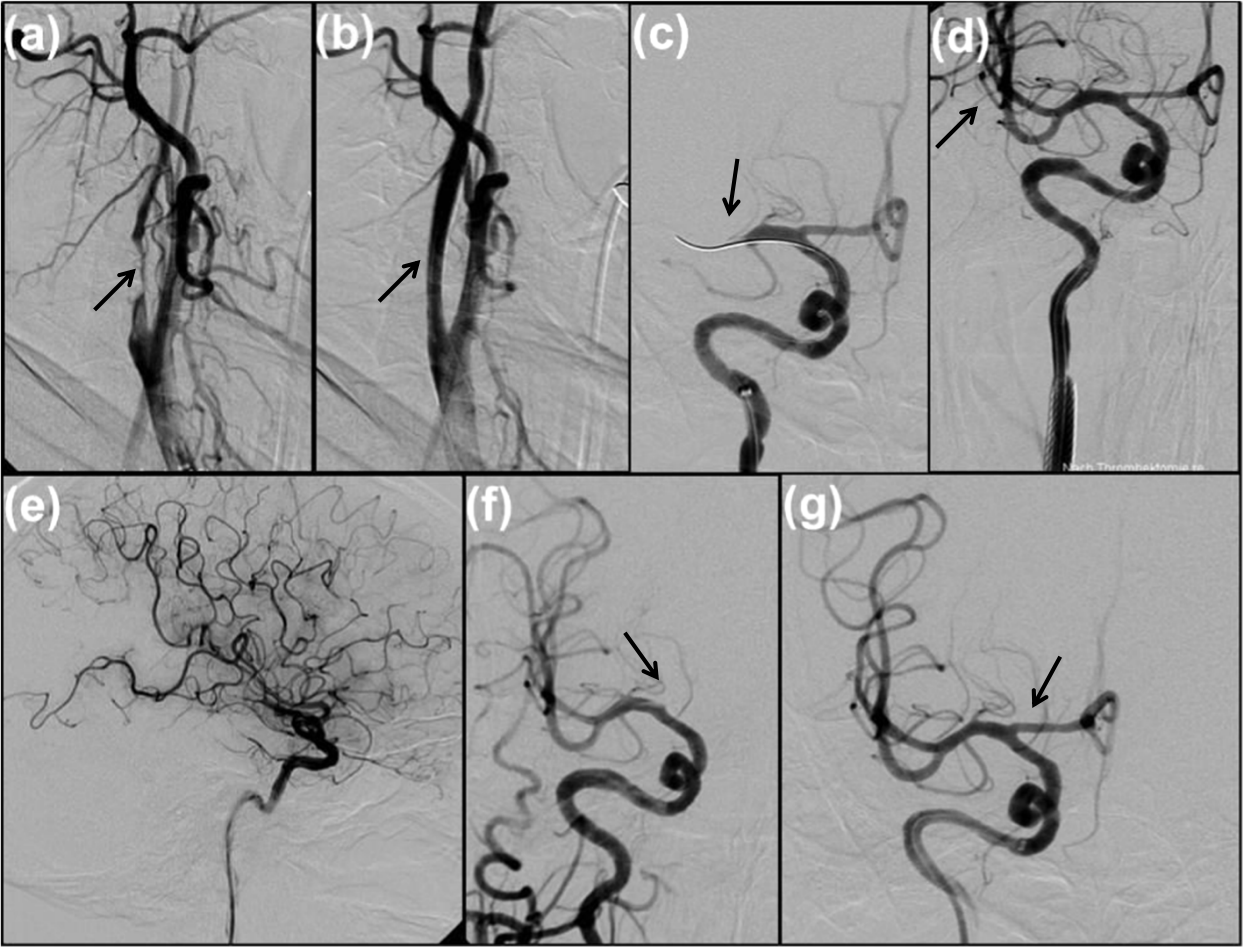
**First angiogram of right internal carotid artery in oblique view demonstrates a high-grade stenosis with calcified and non-calcified parts (a).** Digital subtraction angiography after stent placement (7/50 mm) and balloon dilatation in proximal right internal carotid artery and distal right common carotid artery **(b)**. Recanalization and mechanical thrombectomy maneuver in the right middle cerebral artery **(c)**. Successful reperfusion in M1 to M2 branches of right middle cerebral artery **(d)**. Lateral angiogram of right internal carotid artery shows a residual rarefication of the terminal branches of the right middle cerebral artery in the parieto-occipital region **(e)**. Meanwhile acute occlusion of the A1 segment of right anterior cerebral artery has occurred **(f)**. Final angiogram of the right internal carotid artery at the end of intervention with imaged reperfused M1 and A1 segments and all major vessel branches **(g)**.

Because of the above mentioned conditions, an intravenous systemic thrombolytic therapy was not indicated. Due to the clinical and vascular status and the prominent perfusion mismatch, we decided to perform a neuro-interventional therapy in general anesthesia. We decided not to antagonize anticoagulation because of a considerable risk of developing thrombotic complications during the intervention. Furthermore, continued anticoagulation was estimated to prevent immediate vessel reocclusion after intervention. After initiation of a digital subtraction angiography (DSA) and detection of a filiform stenosis of the proximal RICA (Figure 2(a), 3.50 hours after symptom onset), a stenting of the RICA was performed using a self-expanding wallstent 7/50 mm (Boston Scientific Corp.) followed by balloon dilatation (Figure 2(b)). During the intervention, 500mg of acetylsalicylic acid (ASA) was given followed by continuous body weight-adjusted application of eptifibatide. After confirmation of the proximal M1 occlusion of the RMCA (Figure 2(c)), a mechanical recanalization procedure was performed using the pREset thrombectomy retriever (Phenox, Bochum, Germany) under mild aspiration (Figure 2(d), 4.40 hours after symptom onset). The consecutive angiography displayed a residual rarefication of the parieto-occipital region (see Figure 2(e)), therefore, we performed a superselective DSA of the distal M2 to M3 parts. In doing so, an acute occlusion of the A1 segment of the right anterior cerebral artery was imposed (Figure 2(f), 5.05 hours after symptom onset) followed by an additional mechanical recanalization as described above. Consecutively in the final DSA series, all major vessel branches were opacified (Figure 2(g)). The intervention was terminated 6.30 hours after symptom onset.

**Figure 3:**
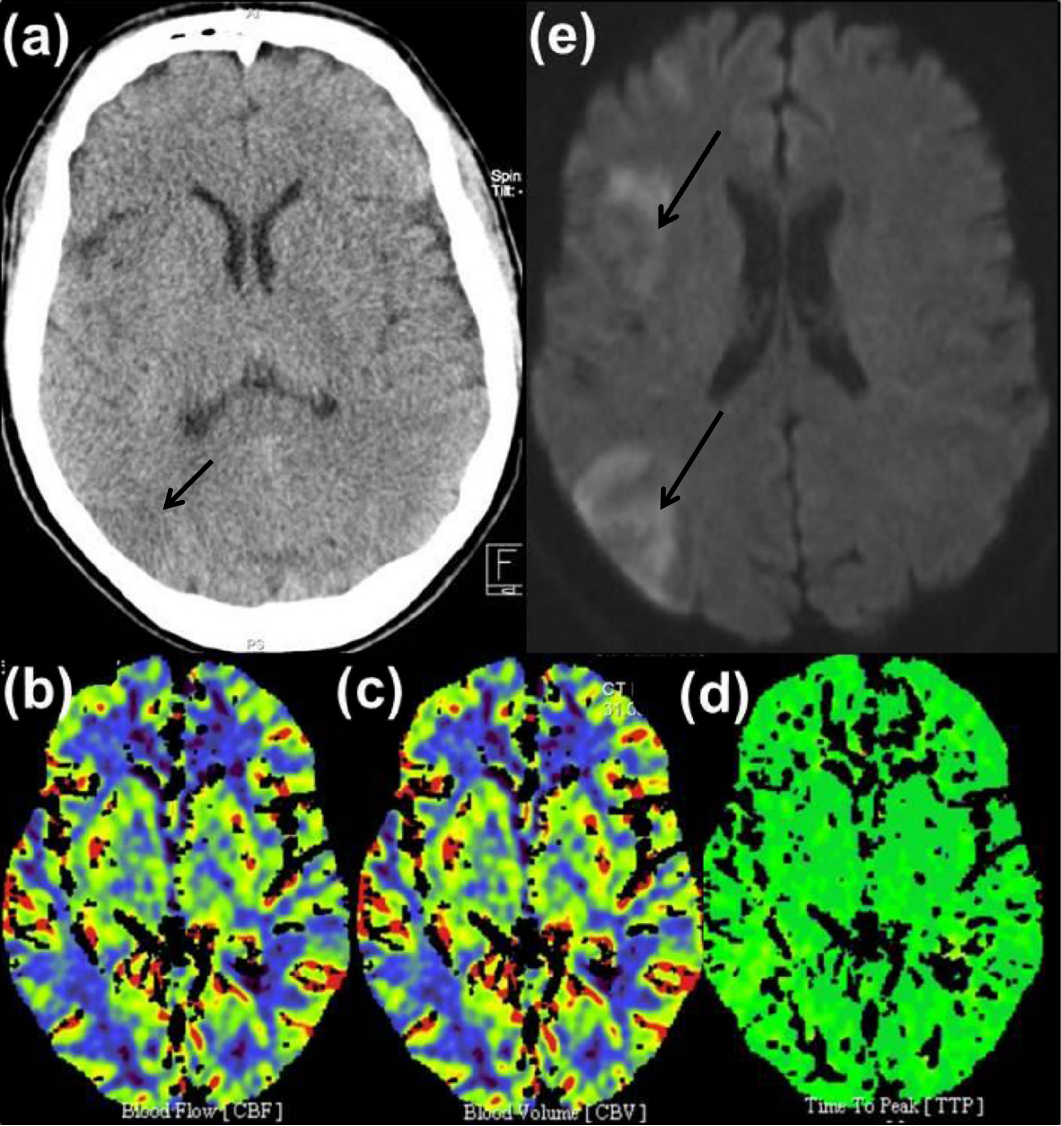
**Native cranial computed tomography one day after treatment (a) and magnetic resonance imaging (diffusion-weighted imaging) 4 days later (e) display limited infarcted areas in right insula and watershed to posterior circulation.** There is neither mismatch nor any perfusion deficit besides the above mentioned limited infarcted areas in the right middle cerebral artery territory in computed tomography perfusion imaging the day after intervention **(b)**-**(d)**.

Automated ventilation could be terminated shortly after the intervention. The patient was admitted to the intensive care unit where he remained clinically stable and conscious presenting with only a low-grade left-sided brachiofacial hemiparesis with a mild dysarthria (NIHSS 4). To prevent stent occlusion, he received an immediate dual platelet inhibition with a clopidogrel loading and continuous oral ASA and termination of eptifibatide. The oral anticoagulation was then antagonized with prothrombin complex concentrate and changed to an intravenous application of heparin because of the anticipated risk of bleeding complications under a combination of platelet inhibition and oral anticoagulation. A cranial CT scan (CCT) 12 hours after recanalization showed a hypodense area parieto-occipitally in his right hemisphere with normal parameter maps for rCBF, rCBV, and mean transit time in CTP (see Figure 3(a)-(d)).

Further work-up (including electrocardiography, ECG; transthoracic echocardiography; transesophageal echocardiography, chest X-ray, 24-hour blood pressure monitoring, 24-hour ECG, sonography of the abdomen, duplex-sonography of the leg arteries and veins, abdominal and chest CT) showed no significant abnormalities. Duplex-sonography of the extracranial and intracranial arteries revealed distinct signs of atherosclerosis. The cranial magnetic resonance imaging 5 days after admission showed small ischemic zones in the RMCA territory (see Figure 3(e)). The screening for thrombophilia revealed pathological results for factor VIII of 209% and for protein S of 50%.

After clinical consolidation, the anticoagulation was again changed to phenprocoumon before discharge. Although an oral anticoagulation with a factor X inhibitor was offered, the patient decided to stay on the vitamin K antagonist. On discharge, he was treated with a combination of ASA 50 mg, clopidogrel 75 mg and phenprocoumon and had only a slight left-sided facial paresis and discrete impairment of motion smoothness in his left hand (NIHSS 2). Apart from that, he was well.

## Conclusions

This case presentation illustrates the methodological advantages of interventional mechanical thrombectomy. First, thrombectomy is not only a promising new complementary therapy in acute stroke, but it can also serve well as an alternative method if intravenous thrombolysis cannot be performed. Second, mechanical thrombectomy can be applied in combination with preceding angioplasty for high-grade extracranial stenosis of the internal carotid artery (ICA). In this patient, it remains unclear whether ICA stenosis was causative for the middle cerebral artery obstruction (since the patient had not been on ASA) or the preexisting factor VIII elevation. In both cases, however, ICA stenosis had to be treated before any other interventional treatment. Angioplasty was favored here. Third, possible complications in the course of the intervention, such as distal embolization of thrombus fragments (in this case anterior cerebral artery obstruction), could be monitored and handled immediately.

The implementation of routine multimodal imaging can help identify patients with contraindications for intravenous thrombolysis which nevertheless could be considered for interventional therapy. Based on native CCT diagnostics alone, patients eligible for a potentially curative therapy might be lost. Therefore, it would be advisable to perform multimodal imaging in all patients with an acute onset of neurological symptoms suspicious of ischemic stroke, even if they have contraindications against an intravenous thrombolytic treatment.

## Consent

Written informed consent was obtained from the patient for publication of this case report and accompanying images. A copy of the written consent form is available for review by the Editor-in-Chief of this journal.

## Abbreviations

ASA: Acetylsalicylic acid; CBF: Cerebral blood flow; CBV: Cerebral blood volume; CCT: Cranial CT scan; CT: Computed tomography; CTA: CT angiography; CTP: CT perfusion imaging; DSA: Digital subtraction angiography; ECG: Electrocardiography; ICA: Internal carotid artery; NIHSS: National Institutes of Health Stroke Scale; RICA: Right internal carotid artery; RMCA: Right middle cerebral artery.

## Competing interests

The authors declare that they have no competing interests.

## Authors’ contributions

KS and JE interpreted the patient data regarding the clinical findings and outcome and were major contributors in writing the manuscript. SS interpreted the patient data regarding the clinical findings. RW and LH interpreted the radiological findings. RW performed the mechanical recanalization. All authors read and approved the final manuscript.
